# Evaluating the impact of social media marketing from the perspective of orthodontists

**DOI:** 10.1186/s12903-024-04558-2

**Published:** 2024-07-11

**Authors:** Eyüp Değirmencioğlu, Hülya Kılıçoğlu

**Affiliations:** Istanbul University Faculty of Dentistry, Department of Orthodontics, Istanbul, Turkey

**Keywords:** Social media, Marketing, Orthodontists, Orthodontic treatment, Post

## Abstract

**Objective:**

In developed countries, orthodontists utilize social media platforms as a pivotal component of their marketing strategies. However, there exists a gap in understanding the broader perspective of healthcare professionals on the utilization of social media in healthcare service delivery. Therefore, this study aims to evaluate the perceptions of healthcare professionals in Turkey regarding the integration of social media within healthcare service delivery.

**Materials & methods:**

This cross-sectional study, conducted between January and February 2023, surveyed 378 members of the Turkish Orthodontic Society. The survey consisted of two parts: a demographic questionnaire with 28 items and a 21-item “Social Media Marketing Activities Scale,” developed with input from three experts. Data analysis will include an explanatory factor analysis. This study provides a snapshot of orthodontists’ perspectives on social media marketing practices.

**Results:**

When participants’ views of patient communication through social media were examined, 19.8% said they “*thought it was right*” and 80.2% said they “*thought it was wrong*”. The treatment and treatment alternatives shared with patients through social media were implemented in 16.5% of cases and not implemented in 83.5% of cases. When examining the social media accounts used by participants to communicate with patients, 56.8% used personal accounts, 43.2% used professional accounts, and when analyzing the social media accounts they used for promotional purposes, 15.8% had personal accounts, 84.2% of them used professional accounts. More than half (*59.8*%) of orthodontists believed that communicating with patients on social media could cause legal problems. The majority of orthodontists (*88.7*%) followed their competitors.

**Conclusion:**

The prevalence of participants’ use of social media posts for advertising purposes was low, and it was determined that the main reason for this was the prohibition of advertising in the provision of health services.

**Supplementary Information:**

The online version contains supplementary material available at 10.1186/s12903-024-04558-2.

## Introduction

Social media platforms have transcended traditional unified communication networks, facilitating seamless interaction and diverse interest sharing among users [1]. Distinguished by their interactive features, social media platforms offer unique advantages over traditional media [2, 3]. Notably, social media has become a cornerstone for marketing various products, offering companies numerous benefits such as brand amplification, cost-effective advertising, sales augmentation, and website traffic enhancement [4]. Moreover, social media utilization is recognized for its potential to cultivate relationships with both prospective and existing customers [5, 6].

Within the realm of healthcare, social media applications have witnessed widespread adoption, particularly within the service sector. Health-related social media tools are commonly leveraged for advertising, promotion, and the dissemination of healthy lifestyle habits. Notably, orthodontists in developed countries have embraced social media as a marketing tool, often showcasing patient treatment photos to engage and attract audiences [7, 8].

Conversely, in Turkey, while social media usage is prevalent, there remains a paucity of research exploring its impact on orthodontic treatment marketing from the orthodontists’ perspective. With the escalating adoption of social media in the country, its potential to serve as a potent patient attraction tool for orthodontists becomes increasingly evident. Consequently, there arises a pressing need to delve into social media marketing from the viewpoint of orthodontists.

In light of these considerations, this study endeavors to meticulously evaluate the impact of social media marketing on orthodontic treatment. We want to assess the methods employed by orthodontists to acquire patients, from the orthodontists’ point of view. We aimed to analyze the content given by orthodontists in their posts to understand the type of content that motivates patients to seek treatment. We inquired about their adherence to the social media profiles of competing clinics. Do you receive assistance from individuals, organizations, or entities to oversee and handle your social media profiles? We inquired about their professional involvement in social media advertising.

By elucidating orthodontists’ perceptions and practices concerning social media marketing, this research aims to furnish valuable insights into harnessing social media’s potential to augment patient engagement and treatment outcomes within the orthodontic domain.

## Materials & method

A total of 378 orthodontists, including 209 males and 169 females, were enrolled in this cross-sectional study, which utilized a retrospective approach based on a survey conducted between January and February 2023. The link to the survey was transmitted to respondents via email. Although the survey was primarily distributed to private practices, it still managed to gather 378 complete responses from the 2031 individuals who were invited to participate. All procedures followed were in accordance with the ethical standards outlined in the Helsinki Declaration of 1975, as revised in 2008 and approved by the Istanbul University institutional ethics committee on November 9, 2021 (protocol number 561). Informed consent was obtained from all participants.

**Fig. 1:**
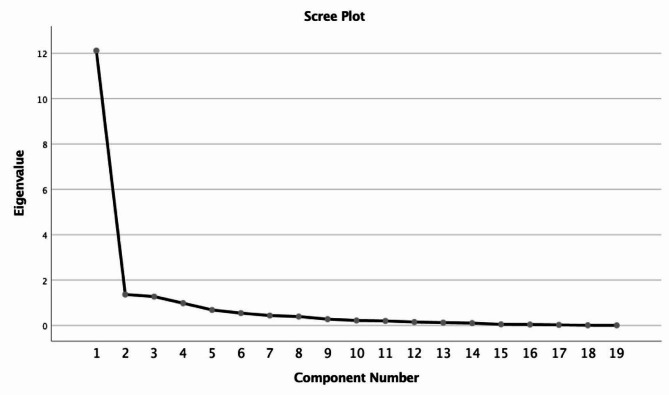
Explanatory factor analysis slope slope plot for the attitude scale towards social media marketing

The survey instrument consisted of two parts: the first part comprised a demographic questionnaire with 28 items designed by the researcher to elicit demographic characteristics of the participating orthodontists and the second part involved the administration of the “Attitude Scale Towards Social Media Marketing,” developed by the investigator to determine orthodontists’ attitudes toward social media use in marketing. Our study was directed to all orthodontists who are members of the association, with the permission of the Turkish Orthodontic Association. Orthodontists in all working areas, such as public hospitals, university hospitals, private practices, private dental clinics, and private hospitals, were included. Exclusion criteria were applied, and general dentists and those specializing in other fields of dentistry were not included in the study. The scale included 19 items measured on a 5-point Likert scale, ranging from strongly disagree (1 point) to strongly agree (5 points). Following data collection, an Explanatory Factor Analysis (EFA) which is used to determine the factor structure during the development stages of the scale was conducted, leading to the removal of 21 social marketing items and two items, as detailed in Fig. 1.

In the EFA application, some values are examined, and whether the scale is suitable for structure is decided. These values are explained in detail below. Kaiser-Mayer-Olkin (KMO) value is used in EFA application to determine whether the sample has a sufficient size for factor analysis, and a KMO value over 0.700 indicates that the sample has a sufficient size for EFA (Aksu et al., 2017: 9; Altunışık et al., 2007: 226). When Bartlett’s test value obtained as a result of EFA is significant, it is interpreted that the scale is suitable for factor analysis and can be divided into factors (Aksu et al., 2017: 10; Altunışık et al., 2007: 230). A slope plot (Screen plot) offers an important prediction in determining the appropriate number of factors. In the slope-slope graph, the graph line becomes linear as it descends vertically. It is stated that it would be appropriate to determine the number of factors equal to the number of points where it changes sharply from vertical to horizontal (Özdamar, 2013: 221; Altunışık et al., 2007: 222; Çokluk et al., 2012: 193). In the EFA application, if a scale item is included in two factors at the same time and there is a difference of less than 0.100 between the factor loadings on the factors it is included in, this item is expressed as an overlapping item. For a mineral to be included in the factor, its factor loading must be at least 0.300 (Altunışık et al., 2007: 226, Çokluk et al., 2012: 194).

### Statistical analysis

The SPSS 22.0 program was used to analyze the data obtained in the research. Frequency analysis was used to determine the percentages of demographic variables. First, exploratory factor analysis (*EFA*) was applied to the scale developed in the research, and a one-sample Kolmogorov-Smirnov analysis was used to determine the reliability and normal distribution. As a result of the analysis, the scale was found to have a single sub-dimension, and the Cronbach’s alpha (α) coefficient was 0.976, indicating a high level of reliability. However, as a result of the one-sample Kolmogorov-Smirnov test, it was determined that the data did not comply with a normal distribution (*p* < 0.05), leading to the decision to use non-parametric tests for comparative analyses. Specifically, Mann Whitney U analysis was utilized to compare scale scores according to variables with two categories, and Kruskal Wallis H analysis was employed for comparisons involving more than two categories.

The population of this study consists of orthodontists who are members of the Turkish Orthodontic Association. Although the number of orthodontists members of the association is 2031, 757 of the members are assistants. The research sample group consisted of 323 orthodontist members selected by a simple random method from among the association members. In the studies in the literature, the number of individuals that should be included in the sample group according to the population size was calculated according to the sample size formula.

## Results

A total of 378 orthodontists were included in this survey analysis. The frequency and percentage distributions of the participants’ demographic information are presented in Table 1. The majority of participants (*70.4%)* used social media. In comparison, 29.6% did not use social media, and when the reasons for not using social media were examined, 36.6% admitted that it is a waste of time, 29.5% admitted that “*I don’t have time*,” 10.7% admitted that “*they don’t use social media and consider it a violation of life*,” 8% admitted that “they don’t know how to use it,” 3.6% admitted that “*it may cause ethical problems*,” 3.6% admitted that “it is not profitable,” and 8% admitted that “*they don’t use social media because of negative content comments*”.

**Table 1:** Frequency and percentage distributions of participants’ demographic information

	Variables	f	%
Gender	Female	169	44.7
	Male	209	55.3
Age group	24–33 years	258	68.3
	34–43 years	89	23.5
	43 ≤ years	31	8.2
Education level / academic title	PhD / Fellows	165	43.7
	Dr / uzman	149	39.4
	Associate Prof. Dr.	46	12.2
	Prof. Dr.	18	4.8
Professional experience	1–5 years	247	65.3
	5–10 years	54	14.3
	10–15 years	36	9.5
	15–20 years	23	6.1
	20 ≤ years	18	4.8
Institution	State Hospital	8	2.1
	University Hospital	248	65.6
	Private Practice	120	31.7
	Private Outpatient Clinic	105	27.8
	Private Hospital	7	1.9

* Since more than one answer can be selected, the total number of answers/percentage may be higher than the total number of participants

The attitudes and behaviors of orthodontists are shaped by the influence of social media on patient preferences, as detailed in Table 2. The proximity of the factor loading to 1, as illustrated in Table 2, signifies a robust correlation between an individual’s marketing attitude and the corresponding measurement scale.

The frequency and percentage distributions of participants’ use of social media for business purposes are shown in Table 3. Regarding how often participants post professionally on social media, 73.3% share once a month or less, 20.7% share once a week, and 6% share 2–3 times a week. The majority of participants (77.4%) answered yes to the question “*Do you communicate with patients on social media*?” and 22.6% answered *no*. Orthodontists’ use of social media was as follows: 99.2% Instagram, 51.1% Facebook, 41.4% Twitter, 66.9% YouTube, 67.3% Google, and 25.2% LinkedIn. Looking at the time spent on social media, 72.6% spent between 1 and 30 min, and 14.3% spent between 30 and 60 min.

**Table 2:** Attitude scale towards social media marketing explanatory factor analysis factor loadings

Attitude Scale Items Towards Social Media Marketing	Factor Load
1) Our clinic’s social media accounts are suitable for sharing content about our services and treatments.	0.736
2) Our clinic is in regular communication with its followers	0.856
3) Our clinic provides precise information about our services and treatments on its social media accounts.	0.770
4) Our clinic’s information on social media accounts about our services and treatments is comprehensive.	0.821
5) The information shared about our services and treatments on our clinic’s social media accounts is current and updated regularly.	0.810
6) Shares made on our clinic’s social media accounts meet the expectations of patients	0.860
7) Our patients’ experiences are shared on our social media accounts.	0.581
9) It is emphasized that we attach importance to patients’ privacy on our clinic’s social media accounts.	0.739
10) Our clinic has useful information for patients on its social media accounts.	0.896
11) Our clinic’s social media posts make appointment suggestions about our services and treatments.	0.658
12) Our clinic’s social media posts about our services and treatments motivate the patient to get treatment	0.879
13) Our clinic’s social media posts contain explanations that will reassure the patient.	0.901
15) Shares on our clinic’s social media accounts meet our promotional needs.	0.774
16) Shares made on our clinic’s social media accounts facilitate information search.	0.849
17) Shares on our clinic’s social media accounts concern current treatments.	0.889
18) I advise my patients to follow and visit our clinic’s social media accounts.	0.741
19) I would like my relatives to recommend our clinic’s social media accounts to their acquaintances.	0.670
20) Social media is an effective medium to inform patients about our new treatments and equipment	0.787
21) Our clinic’s social media accounts increase our patient potential	0.863

**Table 3:** Frequency and percentage distributions of participants’ use of social media for business purposes

Question	Answer	f	%
How often does he post professionally on social media?	1 or less per month	195	73.3
	1 per week	55	20.7
	2–3 per week	16	6.0
Do they communicate with their patients through social media?	Yes	206	77.4
	No	60	22.6
Social media accounts that patients communicate with*	Instagram	184	89.3
	Facebook	30	14.6
	Twitter	6	2.9
	Google	27	13.1
	LinkedIn	15	7.3
Views of patients on social media communication	I find it true	75	19.8
	I find it wrong	303	80.2
Do you talk to patients about treatment and treatment alternatives on social media?	Yes	44	16.5
	No	222	83.5
Social media accounts used to communicate with patients	personal account	151	56.8
	professional account	115	43.2
Social media accounts used for promotional sharing	personal account	42	15.8
	professional account	224	84.2
Which posts by orthodontists on social media attract your attention more? *	Photos of patients	169	63.5
	Photos of clinical staff	12	4.5
	Photos of the orthodontist in the clinic	67	25.2
	Introducing a new product or treatment method	176	66.2
	Photographs or videos taken while the patient is undergoing the procedure	89	33.5
	Photos of the clinic	66	24.8
	Orthodontist participation in assistance programs	30	11.3
	Orthodontist’s participation in public service events	22	8.3
	The private life of the orthodontist (sports, travel, etc.)	71	26.7
For what purposes does he use social media professionally? *	İncrease the number of patients	88	374
	Because my patients use social media	106	45.1
	Because other orthodontists use	86	36.6
	Communicating with my patients via social media	60	25.5
What do patients say they pay attention to when choosing you? *	Coincidence (I saw it passing by etc…)	130	34.4
	Social media account	79	20.9
	Web site	69	18.3
	Institution agreement	59	15.6
	Participating in TV programs	15	4.0
	Familiar advice (Spouse, friend, other physicians)	233	61.6
	Title	98	25.9
	Advice from my patients	227	60.1
	Because I have my practice or clinic	85	22.5
	Location (close to home, in a luxury neighborhood)	83	22.0
	Due to the clean appearance of the clinic and the tools in the clinic	69	18.3
Do patients share intraoral and/or extraoral photographs of patients to promote the business?	Yes	55	20.7
	No	211	79.3

* Total number of answers/percentage ratio may be greater than the total number of participants, as more than one answer can be selected

When the social media accounts used by the participants to communicate with patients were examined, 56.8% of them used personal accounts, 43.2% used professional accounts, and when the social media accounts they used for promotional purposes were analyzed, 15.8% had personal accounts, 84.2% of them used professional accounts. The answers to the question “*Which posts by orthodontists on social media attract more attention?*” were 63.5% photos of patients, 4.5% photos of clinic staff, 25.2% photos of the orthodontist, and 66.2% introducing a new product or treatment method. This was followed by 33.5% photos or videos of the patient undergoing a surgical procedure, 24.8% photos of the clinic, 11.3% the orthodontist’s participation in outreach programs, 8.3% the orthodontist’s participation in public service events, and 26.7% answered as the orthodontist’s personal life (*sports*,* travel*,* etc.).*

Orthodontists reported that 37.4% used social media to attract patients, 45.1% used it because their patients had social media accounts, 36.6% used it because their colleagues did, and 25.5% used it to communicate with patients. Physicians also reported that 20.9% of patients were recruited through social media, 18.3% through a website, 34.4% through chance, and 15.6% through an institutional arrangement. Most new patients were referred by friends and acquaintances (*61.6%).* In addition, 4% of patients applied after seeing the dentist on TV programs, and 25.9% trusted the academic title. It was observed that 22.5% answered because it was my practice or clinic, 22% answered because of the location (*close to home*,* in a luxurious neighborhood*), and 18.3% answered because the clinic and the equipment in the clinic looked clean. Orthodontists responded “*yes*” to 20.7% and “*no*” to 79.3% when asked *if they share intra-oral and/or extra-oral photographs of patients to promote their business.*

## Discussion

When examining the findings regarding the business use of social media by the orthodontists participating in the study, it was found that the frequency of orthodontists using social media for marketing purposes was low, with 73.3% of the participants sharing their professional posts once a month. The speed of patient response to orthodontists’ posts was found to be high. The social media platform where patients respond the fastest is Instagram. According to orthodontists, patients generally prefer a doctor-based recommendation from their friends rather than social media posts. In addition, it has been found that posts about doctors’ opinions are effective in getting patients’ attention. In a study conducted on this topic, it was reported that social media posts promoting the dental clinic were effective in attracting patients’ attention, and the number of patients who chose the clinic based on the recommendation of friends was also high [9].

The research found that the main purpose of orthodontists’ use of social media is based on the idea that social media is widely used among patients. As it is known, in recent years it is noticeable that people have widely used social media applications in the process of purchasing products and services. For this reason, it is stated that companies should pay attention to the use of social media to manage consumer behavior in service delivery [10]. Similar studies indicate that consumers widely use social media in the purchase of services [11, 12] and that social media applications influence consumer behavior [13–16].

It is believed that the orthodontists participating in the research need a higher level of communication with patients through social media. Therefore, they need to use social media more actively in their business. It is thought that the reason for the low level of sharing photos of treatment procedures on social media is that they value patient privacy. Orthodontists have an intense work pace and do not find time to use social media, which leads them to communicate less with patients on social media. On the other hand, studies conducted in various industries show that social media applications have been widely used in customer communication and interaction in recent years [17, 18]. Photographing the course of disease treatment has many medical benefits. However, patients’ rights and bodily privacy should be given importance in medical interventions, and in this context, information and documents related to treatment processes should not be openly shared [19].

According to the orthodontists in the study, the most important social media posts that attract the attention of the patient population are patient photos, promotional posts about new procedures, products, and treatments, and posts from orthodontists in the office. One of the main reasons why orthodontists post is to ensure that patients are informed about their new treatment procedures. Studies in the literature indicate that orthodontists generally aim to raise awareness and inform patients through their social media posts [1]. In recent years, the rationale for the active use of social media in health care has been that social media is advantageous in many aspects of communicating with the community when providing health care services [20].

*Parmar et al.* conducted a study on Facebook use and patient-dentist relationships. A total of 44% of patients said they would like to be contacted by their dentist on social media, while 164/460 (*36%)* patients have searched for their dentist on social media. Social media offers opportunities for dental professionals to improve the efficiency of marketing activities and provide additional services such as online diagnosis and Q&A. However, within the professional context, norms and procedures related to the use of social media in patient-dentist communication remain vague and underdeveloped. Furthermore, *Parmar et al.* indicated that while 74% of dentists agreed that social media friendship is not appropriate, 29% accepted friend requests from their patients on social media [21].

The rapidly developing array of physician-only online communities represents a potentially extraordinary advance in providing physicians with educational and informational resources. These online communities give physicians new control over the information they process, but the use of this social media technology carries some risk [22, 23]. This increased connectivity and blurring of personal and professional boundaries in social media has created new challenges for medical professionalism. Social media has given the public greater access to potentially intimate details of the lives of their healthcare providers. Physicians’ characters have been judged by their online actions, and momentary lapses in judgment have had lasting consequences. While there are many definitions of medical professionalism, most include professional competence, integrity, patient confidentiality, patient welfare, and social justice; upholding these commitments is considered the foundation of public trust [24].

Online technologies present both opportunities and challenges for professionalism. They offer innovative ways for physicians to interact with patients and positively impact the health of communities. However, professionalism and the patient-physician relationship should govern these interactions. Institutions should have policies regarding the use of digital media. Education about the ethical and professional use of these tools is critical to maintaining a respectful and safe environment for patients, the public, and physicians. As patients continue to turn to the Internet for health care advice, physicians should maintain a professional presence and direct patients to reputable sources of information. The use of digital media for non-clinical purposes can affect public perceptions of the profession, especially if questionable content is posted by physicians in their personal use of the Web. Maintaining separate personal and professional identities in Web postings can help avoid blurring boundaries in interactions with patients and colleagues [25].

The power of social media-based advertising dictates that this medium offers significant potential for improving awareness and education among orthodontic providers and patients. Social media, apps, and websites have also been used to positively influence patient behavior and knowledge. Clinicians have a legal and ethical obligation to present information without overemphasizing benefits or excluding harms and should not rely on unsupported claims or poor evidence [26]. The results of the Kruskal-Wallis H test for comparing attitudinal scale scores toward social media marketing according to how orthodontists participating in the research use social media professionally are shown in Table 4.

The analysis of scale scores in relation to income status reveals that orthodontists’ attitudes toward social media marketing exhibit an upward trend as their income levels rise. Upon examination of the Table 5, it becomes evident that the perspectives of orthodontists regarding social media marketing differ significantly based on their monthly income.

It is worth noting that orthodontists who employ social media for promotional intentions predominantly publish content directed towards their patients. (According to their sharing patterns, the nine fashionable posts in the fourteenth question of our survey were divided into two groups and statistically compared. Group A comprises patient-priority postings, which consist of photographs taken of patients during their procedures. Group B comprises orthodontic priority posts, which include images of clinic personnel, the orthodontist himself or herself in the clinic, advertisements for new products or treatment methods, photographs of the clinic itself, the orthodontist’s involvement in charitable programs and public service events, and details regarding the orthodontist’s personal life. Upon examination of the table, it becomes evident that orthodontists who share patient-first posts have a higher attitude towards social media marketing than those who share orthodontist-first posts (*p* > 0.05). However, no statistically significant distinction can be found between the sharing styles of orthodontists participating in the study and their attitude levels toward social media marketing (*p* > 0.05). The results of the Mann Whitney U Test show the comparison of attitude scale scores towards social media marketing based on the sharing styles of orthodontists who participated in the research on social media are shown in Table 6.

**Table 4:** Kruskal Wallis H test results for comparison of attitude scale scores towards social media marketing according to how orthodontists participating in the research use social media professionally

Purposes of use	*N*	Median	SD	x^2^	*p*-value
Advertising	103	3.06	0.985	3.144	0.208
Competition	20	3.43	0.913		
Advertising and competition	118	3.00	1.049		

**Table 5:** Results of the Kruskal-Wallis H test to compare attitude scale scores regarding social media marketing among orthodontists participating in the study, organized by income status

İncome Status	*N*	Median	SD	x^2^	*p*-value	Differences between groups
500–1000 $	16	2,92	,768	9,037	,029	2 < 4
1000–2000 $	32	2,65	1,013			
2000–3000 $	73	3,01	1,055			
3000+ $	120	3,22	,993			

**Table 6:** The results of the Mann Whitney U Test show the comparison of attitude scale scores towards social media marketing based on the sharing styles of orthodontists who participated in the research on social media

Social Media Share type	*N*	Median	SD	U	*p*-value
Patients are priority	148	3,06	1,081	6873,5	,987
Orthodontist priority	93	3,05	,904		

More orthodontic professionals employed in private practices responded to the survey. We believe this is due to apprehensions regarding patient acquisition, advertising, and revenue. It is our contention that orthodontists employed in public institutions lack adequate motivation to engage in social media advertising due to the absence of financial incentives associated with patient acquisition. Our initial objective for this research was to include 500 participants; nevertheless, we managed to analyze a total of 378 orthodontists. In Turkey, the exhibition of advertisements pertaining to health services on digital platforms is subject to certain limitations. We therefore conclude that clinicians who were unaware of social media marketing were not included in the study. The individuals who abstained from participating cited a lack of sufficient knowledge regarding the subject.

## Conclusion

In summary, this study delving into orthodontists’ attitudes toward social media applications in marketing revealed a moderate inclination overall. A nuanced variation was observed in orthodontists’ attitudes towards utilizing social media in marketing, contingent upon their patient attraction methodologies.

The findings underscore the potential for orthodontists to expand their reach and engage a broader customer base through effective utilization of social media in their marketing endeavors. However, it is evident that strategies tailored to individual orthodontists’ patient attraction approaches are essential for optimizing the impact of social media marketing efforts.

Moving forward, strategic integration of social media platforms into orthodontic marketing strategies holds promise for enhancing patient engagement and ultimately improving treatment outcomes within the orthodontic community. Further research and tailored interventions are warranted to unlock the full potential of social media in orthodontic marketing practices.

### Electronic supplementary material

Below is the link to the electronic supplementary material.

Supplementary Material 1

Supplementary Material 2

Supplementary Material 3

## Data Availability

All data generated or analysed during this study are included in this published article [and its supplementary information files].

## Declarations

### Editorial support

QA Executive Consultancy, Ozan Batigun MD, MBA in 2023 have conducted the editorial support of this article. www.QAexeutiveconsultancy.com Ozan.Batigun@outlook.com.
