# Supplementary material for: Evaluating the impact of social media marketing from the perspective of orthodontists
Source: BMC Oral Health. 2024 Jul 11;24:779. doi: 10.1186/s12903-024-04558-2 (PMC11242013; doi:10.1186/s12903-024-04558-2)
Supplement: Supplementary file 3 — Supplementary Material 3 [file 12903_2024_4558_MOESM3_ESM.pdf]

Consent form

I ……………Prof.Dr. Hülya Kılıçoğlu…………………………..…………..... [Name] give my consent for information about myself/my child or ward/my relative (circle as appropriate) to be published in BMC Oral Health , Manuscript ID: 818bebd8-b295-47c3-be34-0c95c3b7e9ed v3.0 , ………

..Eyüp Değirmencioğlu……………………………………………………………………………..………

[Name of journal, manuscript number and corresponding author].

I understand that the information will be published without my/my child or ward's/my relative's (circle as appropriate) name attached, but that full anonymity cannot be guaranteed.

I understand that the text and any pictures or videos published in the article will be freely available on the internet and may be seen by the general public. The pictures, videos and text may also appear on other websites or in print, may be translated into other languages or used for commercial purposes.

I have been offered the opportunity to read the manuscript.

Signing this consent form does not remove my rights to privacy.

Name Prof.Dr. Hülya Kılıçoğlu……………………………………

Date 23.04.2024……………………………………

Signed………………………………………..

Author name Eyüp Değirmencioğlu…………………………..

Date 23.04.2024……………………………………

Signed……………………………………

Please keep this consent form in the patient's case files. The manuscript reporting this patient's details should state that 'Written informed consent for publication of their clinical details and/or clinical images was obtained from the patient/parent/guardian/ relative of the patient. A copy of the consent form is available for review by the Editor of this journal.

BioMed Central Limited, 236 Gray's Inn Road, London WC1X 8HL, UK
BioMed Central Limited is part of Springer Science+Business Media. VAT No. GB 823 8263 26 Registered in England and Wales No. 3680030

T | +44 (0) 20 3192 2000 **F** | +44 (0) 20 3192 2010 **W** | www.biomedcentral.com **E** | info@biomedcentral.com
